# Prolonged ventilation post cardiac surgery - tips and pitfalls of the prediction game

**DOI:** 10.1186/1749-8090-6-158

**Published:** 2011-11-23

**Authors:** Piotr Knapik, Daniel Ciesla, Dawid Borowik, Piotr Czempik, Tomasz Knapik

**Affiliations:** 1 Department of Cardiac Anaesthesia and Intensive Care, Silesian Centre for Heart Diseases, Zabrze, Poland

## Abstract

**Background:**

Few available models aim to identify patients at risk of prolonged ventilation after cardiac surgery. We compared prediction models developed in ICU in two adjacent periods of time, when significant changes were observed both in population characteristics and the perioperative management.

**Methods:**

We performed a retrospective review of two cohorts of patients in our department in two subsequent time periods (July 2007 - December 2008, n = 2165; January 2009 - July 2010, n = 2192). The study was approved by the Institutional Ethics Committee and the individual patient consent was not required. Patients were divided with regard to ventilation time of more or less than 48 hours. Preoperative and procedure-related variables for prolonged ventilation were identified and multivariate logistic regression analysis was performed separately for each cohort.

**Results:**

Most recent patients were older, with more co-morbidities, more frequently undergoing off-pump surgery. At the beginning of 2009 we also changed the technique of postoperative ventilation. Percentage of patients with prolonged ventilation decreased from 5.7% to 2.4% (p < 0.0001).Preoperative and procedure-related variables for prolonged ventilation were identified. Prediction models for prolonged ventilation were different for each cohort. Most recent significant predictors were: aortic aneurysm surgery (OR 12.9), emergency surgery (OR 5.3), combined procedures (OR 5.1), valve procedures (OR 3.2), preoperative renal dysfunction (OR 2.9) and preoperative stroke or TIA (OR 2.8).

**Conclusions:**

Prediction models for postoperative ventilation should be regularly updated, particularly when major changes are noted in patients' demographics and surgical or anaesthetic technique.

## Background

Patients whose ventilation after cardiac surgery is unexpectedly prolonged are prone to a larger number of complications and higher mortality [1]. Additionally, they may present a practical problem for the regular activity of the postoperative intensive care unit.

In the simplest terms, it can be assumed that up to a certain time limit (usually covering a period of 12-24 hours), the duration of mechanical ventilation may depend on unique characteristics, the type of surgical procedure and local protocols within the individual postoperative ICU. In some ICUs, patients are usually extubated very early, even in the operating theatre, while in others - significantly later [2-6]. Attempts to reduce the time of postoperative ventilation may yield medical and financial benefits, however the early risk of hypothermia, bleeding and cardiorespiratory instability may sometimes outweigh the potential benefits [2]. Time of postoperative ventilation depends on many factors [7-9], extubation criteria may vary between departments, and finally - early extubation may be dependent on the technique of anaesthesia [2] or sedation [7].

After the passage of 48 hours, only patients with serious, non-transient issues should remain ventilated. This is often due to general health problems, already present prior to surgery.

EuroSCORE is known to correlate with an extended ICU stay but extended ICU stay may be sometimes not associated with prolonged ventilation [10,11]. Additional differences may be population or learning curve-related [12].

During routine departmental audit we noticed that the percentage of patients with prolonged ventilation, remaining at a fairly constant level in our centre for many years, suddenly in 2009 dropped sharply by half. The same audit revealed, that at the same time we started to operate on more elderly patients (trying to avoid cardiopulmonary bypass if possible) and we also changed the technique of postoperative ventilation. We therefore decided to find out, how the prediction model of prolonged postoperative ventilation change within the same postoperative ICU in two adjacent and relatively short periods of time, when a number of significant changes are observed both in population characteristics and the perioperative management.

In the literature, there are various available models, predicting the occurrence of postoperative complications. They are mainly developed in a single institution, therefore they might not apply to other centers. No attempts have been made, however, to determine why the prediction models sometimes vary so much among themselves and what is the practical solution to this problem. The aim of our study was to examine how the prediction model of prolonged postoperative ventilation can change within the same postoperative ICU in two adjacent and relatively short periods of time, when a number of significant changes were observed both in population characteristics and the perioperative management.

## Methods

We performed a retrospective review of all patients in our prospective departmental adult cardiac surgical database in two subsequent 18-month periods (first cohort: July 2007 - December 2008; second cohort: January 2009 - July 2010). The study was approved by the Institutional Ethics Committee and the individual patient consent was not required. The study was carried out in a tertiary care university hospital.

In our database, patients were classified according as: "coronary" (on pump or off pump), "valve" (single or more, replacement or repair), "combined" ("coronary" + "valve"), "aneurysm" (open repair only), "transplant" and "other" (various procedures, not able to create a homogenous group of at least 20 cases per year).

Patients in the categories "transplant" and "other" and 21 patients who died within 48 hours of surgery were excluded from the analysis. All the remaining 4,357 consecutive patients (2,165 in the first cohort and 2,192 in the second cohort) were included in the analysis.

"Unstable course of the disease" indicated patients with recent deterioration due to their cardiac disease. "Critical preoperative state" was reserved for patients on inotropic, mechanical and/or ventilatory support.

"Non-elective surgery" indicated that patients were required to stay in the hospital but could be planned and operated on within a normal schedule. "Emergency surgery" indicated patients with ongoing refractory cardiac compromise, unresponsive to other forms of therapy except for cardiac surgery. All patients who did not fulfill these definitions were considered to be "elective" (Table 1).

**Table 1:** Comparison of patients' cohorts

Group of variables	Variable	2007-2008 (n = 2165)		2009-2010 (n = 2192)		p
Demographic data and general condition	Age > 65 years	897	(41%)	977	(45%)	**0.04**
	Female gender	711	(33%)	696	(32%)	0.44
	Unstable course of disease	801	(37%)	891	(41%)	**0.01**
	Critical preoperative state	9	(0.4%)	8	(0.4%)	0.78

Circulatory function	CCS class IV	193	(8.9%)	197	(9%)	0.93
	NYHA class III or IV	344	(16%)	464	(21%)	**<0.01**
	Previous PTCA/stent	527	(24%)	562	(26%)	0.32
	Recent MI up to 90 days	395	(18%)	328	(15%)	**<0.01**
	EF < 40%	233	(11%)	267	(12%)	0.14
	Endocarditis	22	(1.0%)	20	(0.9%)	0.72

Co-morbidities	Diabetes	638	(30%)	582	(27%)	**0.03**
	Arterial hypertension	1555	(72%)	1713	(78%)	**<0.01**
	BMI > 35	106	(5%)	99	(5%)	0.78
	Renal failure	46	(2%)	56	(3%)	0.35
	COPD	214	(10%)	193	(9%)	0.22
	History of TIA or stroke	133	(6%)	125	(6%)	0.54
	Carotid disease	248	(12%)	289	(13%)	0.08
	Peripheral vascular disease	312	(14%)	308	(14%)	0.73

Procedure -related variables	Previous cardiac surgery	82	(4%)	78	(4%)	0.69
	Non-elective surgery	338	(16%)	625	(29%)	**<0.01**
	Emergency surgery	62	(3%)	75	(3%)	0.29
	CABG	783	(36%)	412	(19%)	**<0.01**
	OPCAB/MIDCAB	576	(27%)	876	(40%)	**<0.01**
	CPB > 2 h	532	(25%)	444	(20%)	**<0.01**
	Coronary surgery	1359	(63%)	1288	(59%)	**0.01**
	Valve surgery	500	(23%)	577	(26%)	**0.01**
	Combined procedures	206	(9.5%)	218	(10%)	0.63
	Aortic aneurysm surgery	100	(4.6%)	109	(5.0%)	0.58

Patients in both cohorts were then divided into patients ventilated 48 hours or less, and patients ventilated more than 48 hours. In patients who were extubated and later required reintubation, the time of postoperative ventilation was added, which usually qualified them into the "prolonged ventilation" group.

Departmental criteria for extubation included: haemodynamic stability, lack of significant arrhythmias, minimal drainage (<100 mL·h^-1^) and postoperative pain, normal neurological status (absence of neurological deficit and full consciousness), oxygen saturation > 95% with fraction of inspired oxygen <0.5, oesophageal temperature>36.0°C and respiratory rate of more than 12/min. with no signs of respiratory distress. The decision regarding extubation was always undertaken by a physician in charge. Postoperatively, the patients were sedated with either intermittent bolus doses of midazolam or with propofol infusion. Intravenous injections of morphine were used to control postoperative pain.

Preoperative risk assessment was performed on the basis of standard (additive) EuroSCORE and patients were classified as carrying low (0-2 points), moderate (3-5 points) or high risk (6 or more points). Additionally, logistic Euroscore was calculated for each patient since the standard Euro SCORE can underestimate mortality in high-risk patients [13].

Numerical data are shown by mean and standard deviation and compared with Mann-Whitney test. Binary data are shown as numbers and a percentages and compared with the use of the χ^2 ^test with Yates correction, where appropriate. Independent preoperative variables that could affect postoperative ventilation time were identified and are listed in Table 2. The effect of independent variables on the outcome variable of interest (prolonged ventilation) was calculated with the use of multivariate logistic regression. Variables with p value<0.05 were then included in the multivariate logistic regression analysis, where p < 0.05 was considered significant.

**Table 2:** Comparison of all patients with normal and prolonged ventilation

Group of variables	Variable	Normal ventilation (n = 4182)		Prolonged ventilation (n = 175)		p
Demographic data and general condition	Age > 65 years	1780	(43%)	94	(54%)	**<0.01**
	Female gender	1329	(32%)	78	(45%)	**<0.01**
	Unstable course of disease	1594	(38%)	98	(56%)	**<0.00**
	Unstable course of disease	1594	(38%)	98	(56%)	**<0.01**
	Critical preoperative state	8	(0.2%)	9	(5.1%)	**<0.00**
	
	Critical preoperative state	8	(0.2%)	9	(5.1%)	**<0.01**

Circulatory function	CCS class IV	366	(8.8%)	24	(14%)	**0.02**
	NYHA class III or IV	740	(18%)	68	(39%)	**<0.01**
	Previous PTCA/stent	1053	(25%)	36	(21%)	0.17
	Recent MI up to 90 days	694	(17%)	29	(17%)	0.99
	EF < 40%	467	(11%)	33	(19%)	**<0.01**
	Endocarditis	37	(0.9%)	5	(2.9%)	**0.03**

Co-morbidities	Diabetes	1164	(28%)	56	(32%)	0.23
	Arterial hypertension	3149	(75%)	119	(68%)	**0.03**
	BMI > 35	196	(4.7%)	9	(5.1%)	0.78
	Renal failure	92	(2.2%)	10	(5.7%)	**0.01**
	COPD	389	(9.3%)	18	(10%)	0.66
	History of TIA or stroke	235	(5.6%)	23	(13%)	**<0.01**
	Carotid disease	511	(12%)	26	(15%)	0.30
	Peripheral vascular disease	599	(14%)	21	(12%)	0.39

Procedure -related variables	Previous cardiac surgery	142	(3.4%)	18	(10%)	**<0.01**
	Non-elective surgery	922	(22%)	41	(23%)	0.67
	Emergency surgery	104	(2.5%)	33	(19%)	**<0.01**
	CABG	1157	(28%)	38	(22%)	0.08
	OPCAB/MIDCAB	1432	(34%)	20	(11%)	**<0.01**
	CPB > 2 h	868	(21%)	108	(62%)	**<0.00**
	CPB > 2 h	868	(21%)	108	(62%)	**<0.01**
	Coronary surgery	2589	(62%)	58	(33%)	**<0.00**
	Coronary surgery	2589	(62%)	58	(33%)	**<0.01**
	Valve surgery	1026	(25%)	51	(29%)	0.17
	Combined procedures	388	(9.3%)	36	(21%)	**<0.01**
	Aortic aneurysm surgery	179	(4.3%)	30	(17%)	**<0.00**
	
	Aortic aneurysm surgery	179	(4.3%)	30	(17%)	**<0.01**

Outcome	Death	28	(0.7%)	47	(27%)	**<0.01**

## Results

In comparison to the first cohort, patients in the second cohort were more frequently aged over 65 years, with unstable course of their cardiac disease, and more frequently presented signs of congestive heart failure (NYHA class III or IV), while their mode of operation was more frequently urgent. The percentage of coronary revascularization performed off-pump increased from 27% to 40% (p < 0.0001). Percentage of patients with prolonged ventilation decreased from 5.7% to 2.4% (p < 0.0001) (Table 1).

Preoperative status and the distribution of procedures performed in patients with standard and prolonged ventilation differed significantly in the entire population studied (Table 2).

**Figure 1:**
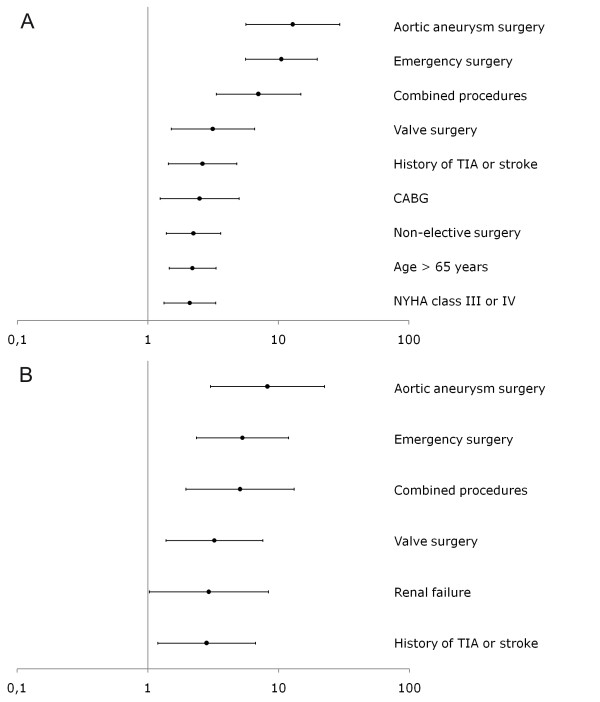
**Multivariate predictors for prolonged ventilation in a first cohort (top figure) and second cohort (lower figure)**.

Multivariate analysis revealed that the independent predictors for prolonged ventilation were different for the two cohorts (Figure 1). The most significant predictors (aortic aneurysm surgery, emergency surgery, combined procedures, valve procedures) remained in their ranking order, however some of the previous significant predictors (age>65 years, NYHA class >2, urgent surgery and CABG) were no longer present in the new model.

**Figure 2:**
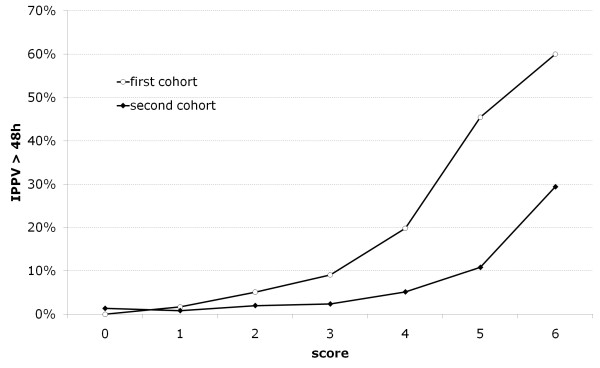
**Scoring system for the first cohort overestimating a risk of prolonged ventilation in a second cohort**.

On the basis of multivariate logistic regression models we developed a scoring scale (in points) assessing the risk of prolonged ventilation for the first cohort. Then, for each group of patients with the same number of points (separately in the first and second cohort) we determined the actual risk of prolonged ventilation and draw the curves of the risk of prolonged ventilation for the different scoring results. As it may be seen, scoring system for the first cohort clearly overestimated a risk of prolonged ventilation, when used in a second cohort (Figure 2).

## Discussion

The results of our study indicate, that our population and patients' management constantly evolves. Our most recent patients are older, have more co-morbidities, and are more frequently undergoing off-pump surgery. In a most recent cohort, significant predictors for prolonged ventilation were aortic aneurysm surgery, combined procedures, valve procedures, preoperative renal dysfunction and preoperative stroke.

Cardiothoracic centres perform a considerable number of highly repetitive procedures. Together with a well organized local database, this may provide a solid basis for the application of various predictive models for all types of postoperative complications (neurological, respiratory, cardiovascular, infectious) or death. The literature abounds in various prediction models for postoperative complications, but their analysis may be quite confusing.

Prolonged ventilation is variably defined. It may be classified as postoperative ventilation continued for more than 12 hours [14], 24 hours [15,16], 48 hours [17], 72 hours [18], or even 96 hours after the operation [19]. Not surprisingly then, the results obtained may be contradictory and of very limited practical applicability.

What is more, authors tend to narrow down the groups of subjects, creating predictive models for prolonged ventilation in patients after one type of procedure only - for example, after coronary revascularisation with cardiopulmonary bypass [14,15], adult valve procedures [16,20],or aortic aneurysm surgery [17]. The only benefit of this approach is the increased homogeneity of the studied group.

Mean extubation times were not the focus of this study. The primary objective was to obtain a practical possibility of predicting a successful execution of a theatre plan taking into account a limited number of intensive beds. It is well known that a patient whose ventilation takes longer than 48 hours, will constitute not only a medical, but also an organizational challenge for the department.

Originally, our intention was to develop a prediction model based on a large population of patients operated on between 2007 and 2010, on the basis of approximately 6,000 patients. Quite surprisingly, however, it turned out that the percentage of patients with prolonged ventilation (>48 hours), which for many years (2004-2008) remained at a fairly constant level (approximately 5%), suddenly in 2009 dropped sharply by half (to 2.5%), reaching values lower than those reported by authors of comparable reports [21]. More interestingly, the characteristics of patients also changed - in a more recent period, there were more elderly patients, with many co-morbitities. The surgical technique also changed - in a subgroup of patients undergoing coronary revascularization, an almost two-fold increase was observed in the percentage of procedures performed without cardiopulmonary bypass (from 37% to 68%). Finally, also in 2009, we changed the technique of postoperative ventilation, introducing a lung protective strategy with higher end-expiratory pressures (PEEP) and smaller tidal volumes for all operated patients, although this method is not aimed primarily at patients with healthy lungs [22]. Tidal volumes were reduced from 7-8 ml/kg to 5-6 ml/kg, whilst PEEP levels were increased from 0-3 cm H2O to 6-8 cm H_2_O. Additionally, patients started to be extubated directly from the ventilator, without a spontaneous breathing trial.

If we had analyzed in detail the differences between the periods of 2007-2008 and 2009-2010, we would probably have identified an even greater number of more or less important differences. This is not surprising, as this phenomenon occurs in every department - medical care continually evolves, changes are made, and the team climbs higher and higher in their skills as per the learning curve principle. The consequence of all these changes, however, is that the prediction models developed for a specific period of time may prove out of date, as was the case with prolonged ventilation. The model developed in 2007-2008 was not very useful for the population of patients operated on in 2009 and 2010.

Comparing the prediction models from 2007-2008 and 2009-2010, we found some very interesting relationships. It turned out that the most important predictors (in order - aortic aneurysm surgery, emergency surgery, complex procedures and valve procedures) remained in their places in the "ranking" of prediction, still significantly increasing the risk of prolonged ventilation. Some of the previous predictors, however, were missing - namely, urgent operation, advanced age (>65 years), presence of congestive heart failure (NYHA III or IV) and coronary revascularization with cardiopulmonary bypass.

The absence of these predictors in the new model may be logically explained. We have probably advanced more in the management of sick, elderly patients with overt heart failure. The percentage of urgent patients has increased from 15.6% to 28.5%, so urgent surgery has become the rule rather than the exception. Coronary revascularization with cardiopulmonary bypass surgery (CABG technique) was once reserved for more difficult coronary patients, whilst more straightforward cases tended to be scheduled for OPCAB. Today, the trend has reversed and the OPCAB technique is now considered suitable for elderly patients with various co-morbidities [23].

In our department we are interested in a realistic prediction of postoperative complications. The ultimate goal is to identify predictors and to apply them in practice - otherwise, it becomes art for art's sake. In accordance with these principles, our department has already introduced prediction models for renal replacement therapy, permanent neurological complications and prolonged ventilation (>48 hours). Each model was developed with a different method.

The primary factor in determining how to conduct a given analysis is always an audit revealing the incidence of complications over the previous few years. Our audit showed that the fractions of patients undergoing renal replacement therapy, and patients who experienced permanent neurological complications, were fairly constant and averaged between 2.5% and 2.7% respectively, whilst the proportion of patients with prolonged ventilation decreased suddenly in the last two years (from 5% to 2.5%).

For the prediction of renal replacement therapy, we re-introduced the 2007 model proposed by Wijeysundera et al., originally encompassing 20,131 Canadian patients [24]. When in 2008, it was concluded that the model worked very well also for the Polish population, it was introduced in our department. The model appears to be reliable, as the incidence of renal replacement therapy remains constant and, additionally, the demographic data of our patients seem to drift slowly towards the Canadian cohort [25].

For the prediction of permanent neurological complications, however, we decided to develop our own model, based on the analysis of our large cohort of 6,016 consecutive patients [26]. We were forced to ignore the existing models proposed in the literature, as they were inconsistent and resulted in conflicting results when tested on our population. We plan to use our model for at least another two years, as the incidence of permanent neurological injury in our department is also fairly constant. Both the above-presented models are likely to be subjected to routine verification in a couple of years, unless the data from internal audits indicate that the prevalence of any of these complications has suddenly changed.

Therefore, in the prediction of prolonged ventilation, we had to choose another solution. We decided to introduce a model based on an analysis of data derived only from the 2009-2010 period (due to the recent sudden decline in the proportion of patients with prolonged ventilation).

What is going to happen next? Does this mean that over the next few years, in predicting prolonged ventilation, we will be using the model based on the analysis of 2,192 patients only?

We do not know yet; the answer depends on the situation! In 2012 we plan to check whether the proportion of patients requiring prolonged ventilation has set on a new, lower level or still continues to change. If this proportion proves to be permanent - we will perform prospective validation of this model in a most recent population. If satisfactory, we will recalculate the data for a larger population (and thus for the entire period from 2008 to 2012) and to make the necessary corrections (which are likely to be minor). Otherwise, we will have to develop another model based on the previous two years ... unless, in the meantime, the medical literature offers us a better solution to this problem.

An important limitation of our study is in the retrospective data extraction, however a lot of work is carried out to confirm the high quality of the hospital database. Another limitation of the study was the exclusion of all patients in the categories "transplant" and "other", but it is always very difficult to create predictive models on the basis of such non-homogenous groups.

The results of our study indicate, that prediction game is particularly prone to bias and misinterpretation and prediction models should be always approached with caution. Moreover, even centre-specific, departmental prediction models should be updated, when major changes are noted in patients' demographics or management.

## Competing interests

The authors declare that they have no competing interests.

## Authors' contributions

PK conceived and planned the study and drafted an early, initial olan of the manuscript manuscript.

DC planned and performed statistical analysis, drafted specific part of the manuscript (figures, legends, statistical analysis).

DB performed literature search, drafted specific part of the manuscript, participated in the design and coordination.

PC performed extensive literature search and drafted specific part of the manuscript.

TK performed extensiveliterature search and drafted specific part of the manuscript.

All authors read and approved the final manuscript.
